# Synthesis, Characterization, Crystal Structure, and DFT Study of a New Square Planar Cu(II) Complex Containing Bulky Adamantane Ligand

**DOI:** 10.3390/molecules23030701

**Published:** 2018-03-20

**Authors:** Monther A. Khanfar, Areej M. Jaber, Murad A. AlDamen, Raed A. Al-Qawasmeh

**Affiliations:** Department of Chemistry, Faculty of Science, the University of Jordan, Amman 11942, Jordan; areejmajed@ju.edu.jo (A.M.J.); maldamen@ju.edu.jo (M.A.A.); r.alqawasmeh@ju.edu.jo (R.A.A.)

**Keywords:** 2-furyl adamantanhydrazone, hydrazone complexes, square planar copper(II) complex, DFT

## Abstract

A copper complex with square planar geometry, [(L)CuBr_2_] (**1**), (L = *N′*-(furan-2-ylmethylene)adamantne-1-carbohydrazide) has been synthesized and characterized by Fourier transfer infrared (FTIR) spectroscopy, elemental analysis, mass spectrometry, and single crystal X-ray diffraction. The crystal of **1** is solved as monoclinic, space group P2_1_/m with unit cell parameters: *a* = 10.8030(8), *b* = 6.6115(8), *c* = 12.1264(12) Å, *β* = 101.124(8)°, V = 849.84(15) Å^3^, Z = 2, and R_1_ = 0.0751 with wR_2_ = 0.1581 (I > 2 σ). The structure of **1** shows intramolecular hydrogen bonding between the N–H and the furan oxygen which stabilizes the configuration of the complex. Furthermore, inside the lattice there are other weak interactions between bromo ligands and the ligand L. DFT calculations where performed to study the stability of this geometry.

## 1. Introduction

Hydrazones constitute an important class of compounds and have been extensively studied due to their rapid access, availability, and diversity. Incorporation of a carbonyl group to hydrazones forms the acrylhydrazones, which in turn have an additional ligating site. Compounds of this type were accessed due to their biological significance as, for example, antifungal [1,2] antimicrobial [3,4,5,6,7,8,9], and anticancer [10,11,12] agents. Furthermore, an adamantane moiety has been known to enhance biological significance when anchored on certain scaffolds [13,14,15,16,17,18]. On the other hand, numerous instances of metabolism and transportation of metal ions and complexes have been established, as well as the importance of introducing adamantane on metal ion-containing hydrazones, yet less attention was devoted to such compounds [19,20,21,22]. Copper is considered to be an essential trace element in biological systems [23,24,25]. The importance of the coordination of copper with ligands containing adamantane [26] has also been investigated. Recently, copper complexes as a substitute of cisplatin complexes as anticancer agents have been reviewed [27,28]. Among the variety of coordination numbers and geometry that copper could deliver, the square planar geometry would be most attractive. Nevertheless, incorporation of metal ions with biologically active ligands may not enhance the activity. This may be attributed to structural changes that the metal ion could make in the structure of the organic ligand [29]. It is still a challenging task to collect active functional groups in a compound, especially for drugs [29]. Quite recently, we reported the synthesis of new adamantane-containing hydrazone compounds, where furan was one of the arms within the hydrazine-adamantyl compounds [30,31]. Herein, we report on the synthesis and study of the structural properties of copper(II) complex of *N′*-(furan-2-ylmethylene)adamantne-1-carbohydrazide which may possess a potent anticancer activity. Interestingly, the synthesized complex showed a symmetrical square planar geometry as indicated by X-ray single crystal crystallography.

## 2. Results and Discussion

### 2.1. Preparation and Characterization of the Copper(II) Complex

**Scheme 1:**
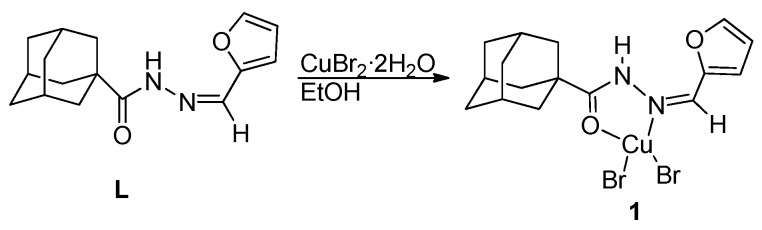
Synthesis of copper(II) complex **1**.

The ligand was prepared as described [30,31] by refluxing an equimolar mixture of admantane-1-carboxylic acid hydrazine with furane-2-caebaldehyde in ethanol to produce the desired ligand, which was identified with an authentic sample [30,31]. The reaction of L with copper(II) bromide (CuBr_2_) under the solvothermal condition results in the formation of Cu(II) complex (Scheme 1). High resolution mass spectrometry (HRMS) of the complex gave the corresponding exact molecular mass. IR bands υ (cm^−1^) assigned to stretching (C=O) at 1593 (s) cm^−1^ and (C=N) at 1526 (s) cm^−1^ clearly shows the weakness of the carbonyl group as well as C=N upon complexation.

### 2.2. Description of the Crystal Structure

**Figure 1:**
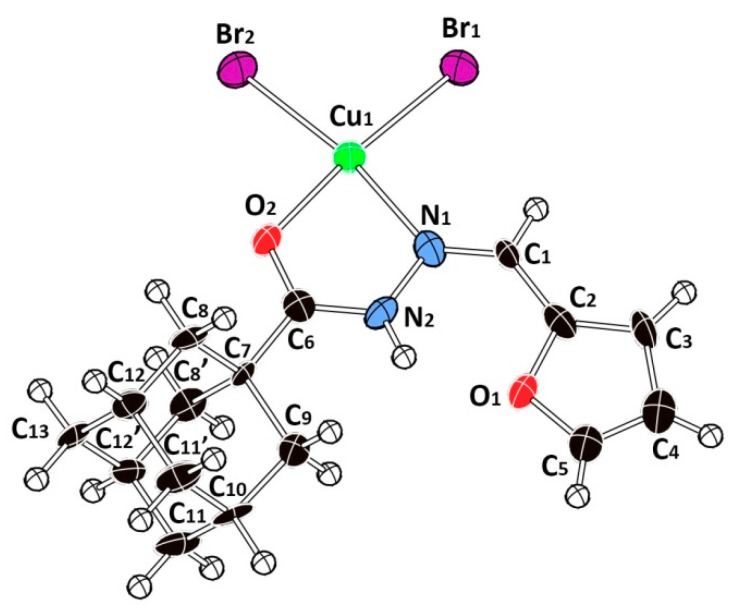
Thermal ellipsoid drawing (50% probability level) of the asymmetric unit of **1**.

Single X-ray crystallography measurements show that [C_16_H_20_Br_2_CuN_2_O_2_] (**1**) crystallizes in the monoclinic system, with space group P2_1_/*m* and crystallographic data listed in Table 1. The asymmetric unit of **1** (Figure 1) contains one molecular unit.

**Figure 2:**
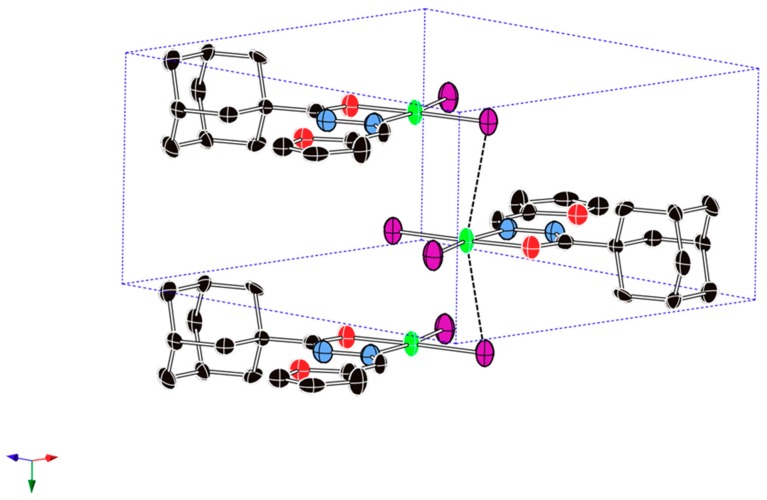
Crystal packing along the c-axis of **1** shows the distorted octahedron around copper, the thermal ellipsoid drawing of 50%.

**Figure 3:**
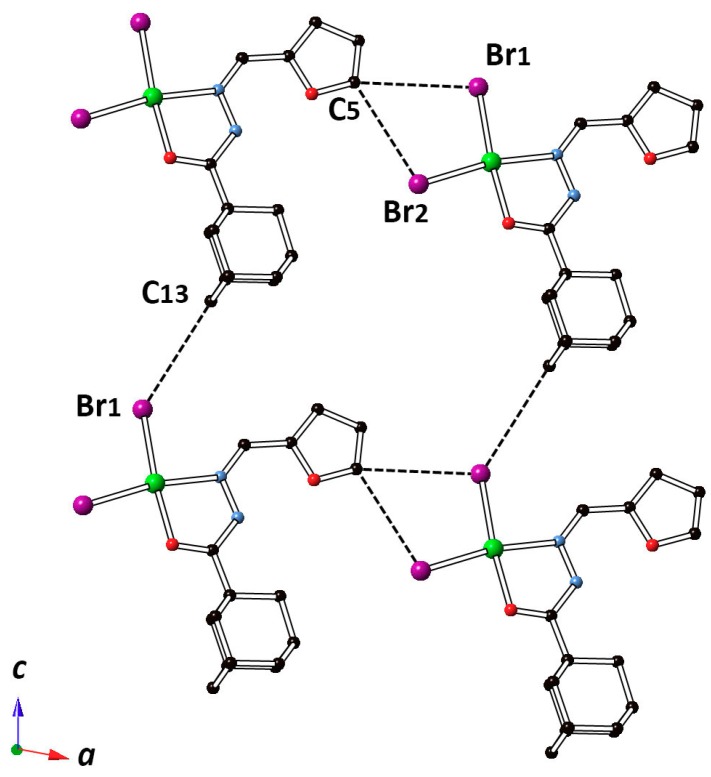
Crystal packing of **1** in the ac-plane, dashed lines is the shortest weak interactions in **1**.

The coordination sphere of copper(II) consists of two bromides with an average Cu_1_–Br bond length of 2.372 Å, a hydroxyl oxygen Cu_1_–O_2_ of 1.987 Å, and Cu_1_–N_1_ of 2.067(10) Å. The Cu(II) is a square planar with Br_1_–Cu_1_–Br_2_ close to 90° (97.03(7)°). The furan and tertiary carbon in the adamantyl (C_7_) group and the amide arm (N_2_ and C_6_) are aligned in a coplanar disposition. All bond lengths and angles are in agreement with those reported for similar copper(II) complexes [32,33,34]. The structure of crystal **1** is stabilized by the presence of hydrogen bonds between the bromine atoms and the L ligands (N_2_-H···O_1_, 2.665(2) Å). Figure 2 shows the packing of **1** and Figure 3 describes the 1D-layer formed from these weak interactions in **1**.

### 2.3. DFT Calculations

**Figure 4:**
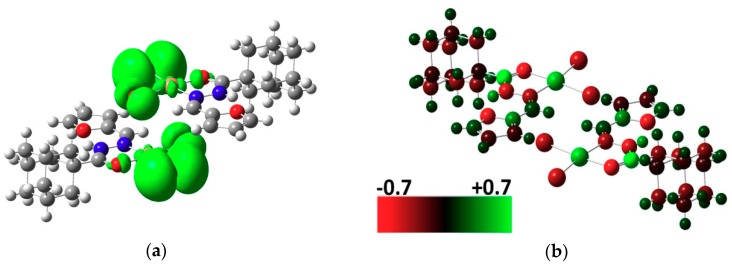
(**a**) ESP (electrostatic potential); and (**b**) Mulliken charge calculations at level 6-31G(d)/B3LYP for the dimer of **1**.

**Figure 5:**
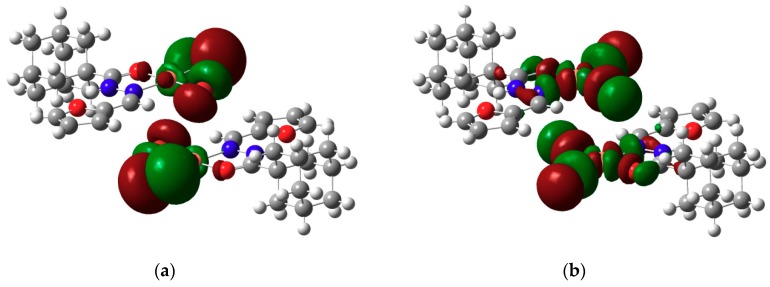

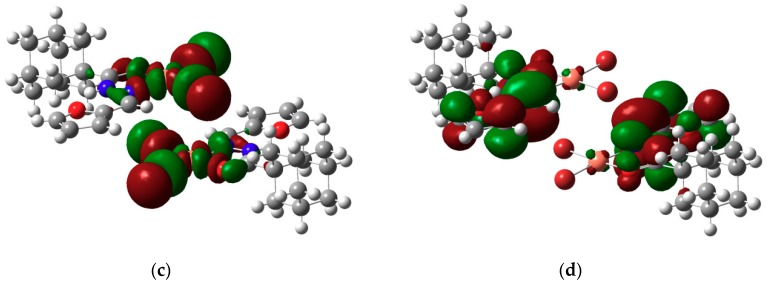
Presentation of the HOMO–LUMO in **1** obtained at 6-31G(d)/B3LYP of theory. (**a**) HOMO-1; (**b**) HOMO; (**c**) LUMO; (**d**) LUMO+1.

The stability of the square planar geometry in **1** was studied by DFT (6-31G(d)/B3LYP) calculations. Overall, we have designed two different models; one is built by enforcing the coordination around the copper atom to be square planar (SP), and the other as a distorted tetrahedron (DT). In the case of SP, the calculations lead to many negative vibration modes. This means unstable optimization with no global minima. The optimization of the monomer with a copper as DT was ended with no imaginary or negative modes. This means that the coordination of the monomer molecule would not be SP. This concludes that the in silico (gaseous) structure is not in agreement with the solid-state structure of **1** (Table 2) which is SP. A literature survey of LCuBr_2_ complexes shows that many systems have been reported with distorted geometry and only a few possess square planar geometry [35,36,37]. Therefore, we optimized a dimer (Figure 4 and Figure 5) to try mimicking the crystallographic structure. The results obtained indicated that there are no negative or imaginary modes and the copper coordination was almost square planar. These findings may indicate that the stability of such a type of coordination system might be due to the interactions (even very weak) in the solid state in **1**. Figure 3 shows CH···Br intermolecular interaction in **1** with C···Br (3.776 Å) and C···H···Br (157.73°). Furthermore, Mulliken and ESP charge calculations show strong interactions between Br_1_ (−0.4) from a monomer with the Cu_1_ (+0.5) (Table 2) from others along b-axis which stabilize the square planar coordination (Figure 4). This could be due to the highly distorted octahedron. To confirm the possibility of having a distorted octahedron, we examined the frontier molecular orbitals and found that no molecular interactions were observed (Figure 5). Such findings unequivocally confirm that, in such complex, copper adopts a square planar geometry, which is rarely described in literature.

## 3. Materials and Methods 

FTIR spectra were recorded with a Nicolet Impact 400 Fourier transform infrared Spectrophotometer (Madison, WI) in the 400–4000 cm^−1^ region. KBr discs for solid samples were made by grinding 2 mg of the solid sample with about 0.2 g of KBr. A background spectrum was subtracted. Mass spectrometry experiments were performed in the negative mode on mass spectrometer (APEX-4 (7 Tesla), Bruker Daltonics, Bremen, Germany) equipped with an ESI source.

### 3.1. Synthesis of the Copper(II) Complex **1**

The copper(II) complex of *N′*-(furan-2-ylmethylene)adamantne-1-carbohydrazide ligand (L) was prepared by the addition of a hot ethanol solution of the previously reported ligand (L) by our group [30,31] to an equimolar amount of copper(II) bromide [38]. The complex was precipitated immediately during stirring of the reaction mixture on a magnetic stirrer at room temperature. The precipitate was filtered, washed with cold ethanol, and dried at 60 °C in a vacuum oven for 2 hours to give the copper complex. Orange block-like crystals suitable for X-ray analysis were obtained: characteristics FTIR (KBr): *ν* = 1593 (s), 1526 (s), 525 (m). ESI-HRMS (*m*/*z*): 491.91148 calculated for C_16_H_19_Br_2_CuN_2_O_2_ [M − H]^−^.

### 3.2. X-ray Difraction Study

Single-crystal X-ray diffraction data were collected using an Oxford Diffraction XCalibur, equipped with (Mo) X-ray Source (λ  =  0.71073 Å) at 291(2) K. Data collection, reduction, and cell refinement were performed using the software package CrysAlisPro [39]. Analytical absorption corrections were applied using spherical harmonics implemented in SCALE3 (ABSPACK) scaling algorithm. Crystal structure was solved by direct methods, using the program OLEX2, followed by Fourier synthesis, and refined on *F*^2^ with SHELXL-97 [40]. Anisotropic least-squares refinement of non-H atoms was applied. All crystallographic plots were obtained using the CrystalMaker program [41]. A summary of the crystallographic data and structure refinement parameters is given in Table 1. Crystallographic data (excluding structure factors) for the structures in this paper have been deposited with the Cambridge Crystallographic Data Centre, CCDC, 12 Union Road, Cambridge CB21EZ, UK. Copies of the data can be obtained free of charge on quoting the depository number CCDC-1824898 (Fax: +44-1223-336-033; E-Mail: deposit@ccdc.cam.ac.uk, http:// www.ccdc.cam.ac.uk). Selected bond lengths and angles are listed in Table 2.

### 3.3. Computational Details

B3LYP density functional theory (DFT) calculations were performed using the Gaussian 09 with its visual interference Gaussview 5 [42]. Due to the issues related to cost, 6-311G(d) basis sets were used for C, H, N, O, Br, and Cu. No symmetry constraint was imposed in the optimization. The vibration analyses were carried out to insure the non-existence of any negative/imaginary modes.

## 4. Conclusions

A new copper complex with *N′*-(furan-2-ylmethylene)adamantne-1-carbohydrazide is prepared and its crystal structure was determined. The coordination of the copper(II) is square planar which is rare. We carried out a DFT calculation and show that this coordination is not stable in (in silico) the gaseous phase. The stability of such coordination in the solid state may be attributed to the existence of weak intermolecular forces between Br and CH.

## Figures and Tables

**Table 1:** Crystal data and structure refinement parameters for **1**.

Crystal System	Monoclinic
T/K	293(2)
Space group	P2_1_/*m*
*a*/Å	10.8030(8)
*b*/Å	6.6115(8)
*c*/Å	12.1264(12)
*β*/°	101.124(8)
Volume/Å^3^	849.9(3)
Z	2
ρ_calc_ g/cm^3^	1.937
μ/mm^−1^	5.997
F(000)	490
Radiation	MoK_α_ (λ = 0.71073)
2Θ range for data collection/°	6.848–51.348
Index ranges	−13 ≤ h ≤ 13, −5 ≤ k ≤ 8, −13 ≤ l ≤ 14
Reflections collected	3640
Independent reflections	1754 [*R*_int_ = 0.0842, *R*_sigma_ = 0.1264]
Data/restraints/parameters	1754/0/130
Goodness-of-fit on *F*^2^	1.017
Final *R* indexes [I ≥ 2σ (I)]	*R*_1_ = 0.0751, *wR*_2_ = 0.1581
Final *R* indexes [all data]	*R*_1_ = 0.1214, *wR*_2_ = 0.1902
Largest diff. peak/hole / e Å^−3^	1.12/−1.07

**Table 2:** Selected bond distances (Å) and bond angles (°) for **1**. Experimental, optimized dimer and monomer structural data are presented.

	Exp.	Theo.			Exp.	Theo.	
		dimer	monomer			dimer	monomer
Cu_1_-Br_1_	2.376(2)	2.379	2.287	C_7_-C_6_	1.505(15)	1.521	1.935
Cu_1_-Br_2_	2.368(2)	2.323	2.298	C_7_C_8_	1.519(10)	1.556	1.556
Cu_1_-O_2_	1.987(8)	2.094	2.036	C_7_-C_9_	1.574(13)	1.548	1.556
Cu_1_-N_1_	2.067(10)	2.094	2.061	C_4_-C_5_	1.307(17)	1.364	1.364
O_2_-C_6_	1.268(13)	1.241	1.243	C_4_-C_3_	1.460(17)	1.424	1.423
O_1_-C_2_	1.385(14)	1.382	1.382	C_2_-C_3_	1.351(17)	1.373	1.375
O_1_-C_5_	1.364(14)	1.362	1.361	C_8_-C_12_	1.521(11)	1.545	1.545
N_2_-N_1_	1.381(13)	1.376	1.372	C_10_-C_9_	1.515(16)	1.541	1.541
N_2_-C_6_	1.320(15)	1.357	1.364	C_10_-C_11_	1.518(11)	1.541	1.543
N_1_-C_1_	1.250(14)	1.295	1.294	C_13_-C_12_	1.526(10)	1.542	1.543
C_1_-C_2_	1.457(16)	1.433	1.432	C_12-_C_11_	1.510(9)	1.542	1.542
Cu_1_···Cu_1_	3.985	3.258	-	N_2_···O_1_	2.665	2.755	2.768
Cu_1_···Br_1_	3.385	2.623	-	N_1_-Cu_1_-Br_1_	93.7(3)	93.39	96.55
Br_2_-Cu_1_-Br_1_	97.03(7)	93.50	109.09	N_1_-Cu_1_-Br_2_	169.3(3)	156.42	138.04
O_2_-Cu_1_-Br_1_	173.9(2)	163.45	147.02	Br_1_···N_1_N_2_C_6_O_2_	0.000	−0.012	−0.538
O_2_-Cu_1_-Br_2_	89.0(2)	90.45	95.43	Br_2_···N_1_N_2_C_6_O_2_	0.000	−0.028	2.109
O_2_-Cu_1_-N_1_	80.3(4)	77.04	77.93	Cu_1_···N_1_N_2_C_6_O_2_	0.000	0.277	0.355
